# Knowledge of danger signs during pregnancy and subsequent healthcare seeking actions among women in Urban Tanzania: a cross-sectional study

**DOI:** 10.1186/s12884-017-1628-6

**Published:** 2018-01-03

**Authors:** Beatrice Mwilike, Gorrette Nalwadda, Mike Kagawa, Khadija Malima, Lilian Mselle, Shigeko Horiuchi

**Affiliations:** 1 Muhimbili University of Health and Allied Sciences, School of Nursing, P.O. Box 65004, Dar es salaam, Tanzania; 2 Department of Nursing, Makerere University, P.O. Box 7072, Kampala, Uganda; 3 Department of Obstetrics and Gynecology, Makerere University, P.O. Box 7072, Kampala, Uganda; 4 Tanzania Commission for Science and Technology, Dar es Salaam, Tanzania; 5 St. Luke’s International University, 10-1 Akashi-cho, Chuo-ku, Tokyo, 104-0044 Japan

**Keywords:** Knowledge, Danger signs, Pregnancy, Healthcare seeking action

## Abstract

**Background:**

Tanzania is among the countries with a high maternal mortality ratio. However, it remains unclear how information and education on danger signs of pregnancy translate into appropriate actions when a woman recognizes danger signs. This study aimed to determine women’s knowledge of obstetric danger signs during pregnancy and their subsequent healthcare seeking actions.

**Methods:**

The study design was a health facility-based cross-sectional study. Quantitative data were collected through interviewer-administered questionnaires. Descriptive and inferential statistics were used to analyze the data. The study enrolled 384 women from two health centers in Kinondoni Municipality, Dar es Salaam, Tanzania. A woman who had not mentioned any danger sign was categorized as *having no knowledge*, mentioned one to three danger signs as *having low knowledge*, and mentioned four or more danger signs as *having sufficient knowledge*.

**Results:**

Among the 384 participants, 67 (17.4%) had experienced danger signs during their pregnancy and reported their healthcare seeking actions after recognizing the danger signs. Among those who recognized danger signs, 61 (91%) visited a healthcare facility. Among the 384 participants, five (1.3%) had no education, 175 (45.6%) had primary education, 172 (44.8%) had secondary education, and 32 (8.3%) had post-secondary education as their highest educational levels. When asked to spontaneously mention the danger signs, more than half of the participants (*n* = 222, 57.8%) were able to mention only one to three danger signs. Only 104 (31%) had correct knowledge of at least four danger signs and nine (2.7%) were not able to mention any item. The most commonly known pregnancy danger signs were vaginal bleeding (81%); swelling of the fingers, face, and legs (46%); and severe headache (44%). Older women were 1.6 times more likely to have knowledge of danger signs than young women (OR 1.61; 95% CI 1.05-2.46)”.

**Conclusion:**

Women took appropriate healthcare seeking action after recognizing danger signs during pregnancy. However, the majority had low knowledge of pregnancy danger signs. Additional studies are warranted to address the knowledge gap and to plan interventions for improving health education under limited resource settings.

## Background

In 2013, about 289,000 women across the world were reported to have died from pregnancy and childbirth-related complications [1]. It is estimated that the majority (62%) of global maternal deaths occur in Sub-Saharan Africa [1]. A high maternal mortality ratio usually characterizes most countries within the Sub-Saharan region, one of which is Tanzania at 556 deaths per 100,000 live births [2]. Other countries in the East African region with a high maternal mortality ratio include Kenya (510/100,000 live births) and Uganda (343/100,000 live births) [3]. The major complications that account for 80% of all maternal deaths are severe bleeding, infections, high blood pressure during pregnancy, obstructed labor, and unsafe abortion. However, many maternal deaths can be prevented if appropriate action is taken early and promptly.

Tanzania’s ongoing efforts to improve maternity care has resulted in the adoption of the World Health Organization’s focused antenatal care (FANC) program consisting of only four visits for low-risk pregnancy without complications. This version of an antenatal care (ANC) program included health promotion, prevention, detection, and treatment of existing diseases. It contained critical information for birth preparedness including the seven danger signs of pregnancy [4].

Every woman needs to be aware of the danger signs that occur during pregnancy, as complications can be unpredictable. These danger signs include vaginal bleeding, severe headache, vision problems, high fever, swollen hands/face, and reduced fetal movement [4]. These danger signs usually indicate the presence of an obstetric complication that may arise during pregnancy, delivery or postdelivery. Knowledge of these danger signs will help women to make the right decisions and take appropriate healthcare seeking actions [5]. Eventually, taking the right healthcare seeking action means receiving immediate and appropriate care, which reduces maternal mortality and morbidity. Therefore, women should receive health education about pregnancy including outcomes, danger signs during pregnancy, nutrition and family planning, as well as other services when they visit an ANC clinic [2].

The 2011 demographic health survey report of Tanzania [2] showed that only 53% of pregnant women were informed about the danger signs of pregnancy during their ANC visits. Other studies have also identified women’s lack of knowledge of these danger signs [5–12]. However, the healthcare seeking actions of women after recognizing a danger sign during pregnancy have not yet been investigated. As all pregnant women are at risk of developing pregnancy-related complications, education on danger signs of pregnancy should be provided to all women who are attending an ANC clinic [3].

During a visit to a clinic, women receive an antenatal card wherein all the services provided during each visit are recorded [4]. However, the antenatal card does not usually include information on danger signs, thus such information may be missed during a visit [11, 12]. Women are advised to go to a nearby health facility and seek care in case they experience any pregnancy danger signs; however, visiting traditional healers, friends, or relatives before going to a health facility is also apparent [13]. In addition, women in South Africa [14] weighed the expected benefits against the anticipated costs before making a decision to avail of healthcare. Thus, travel time and perceptions of staff receptivity were also influential in their decision-making.

However, there are apparently no studies that have found a link between women’s knowledge of danger signs during pregnancy and their subsequent healthcare seeking action if they recognize a danger sign. In this study, we assessed the knowledge of danger signs of pregnant women in Tanzania and their subsequent healthcare seeking action after recognizing the danger signs.

## Methods

### Study design

The study design was a health facility-based cross-sectional study whereby data were gathered at one point in time in a clinical setting. The researcher interviewed the participants using a Swahili questionnaire.

### Study setting

The study was performed in two health centers in Kinondoni municipality, an urban district located in the Dar es Salaam region, Tanzania. The total population of this municipality is 1,775,049 (914,247 women and 860,802 men) according to the 2012 national census report [15]. The maternal mortality ratio of this municipality was 529/100,000 live births [16]. At the time of data collection, there were two health centers available, and data were collected at the Reproductive and Child Health Clinic (RCHC) of these health centers from May 2013 to June 2013. These health centers provide reproductive and child health services as well as maternity services for women who attend the clinic. Normally, antenatal education is provided in the form of a group session with women who have attended the clinic on a particular day. The total number of women who receive care at the RCHC ranged from 60 to 100 women per day. Nurse-midwives provide information about nutrition, birth preparations, obstetric danger signs, and vaccinations following uniform guidelines for providing information according to the available FANC guidelines in the country. Women are advised to visit a nearby health facility for care when they recognize a danger sign during their pregnancy.

### Study participants

Potential participants were 392 postpartum women who were seeking immunization services for their children in May and June 2013. The participants were selected by proportionate systematic random sampling. A woman who had given birth within the past 6 weeks from the day of data collection was eligible for the study.

### Instrument

We developed a questionnaire based on a previous questionnaire about awareness of danger signs among rural women in a study conducted in Tanzania [5]. The questionnaire was translated from English to Swahili, which is a language most familiar to Tanzanians, by a trained research assistant and an experienced midwife. The questionnaire is composed of four sections: socio-demographic characteristics, experiences in the last pregnancy, knowledge of pregnancy danger signs, and healthcare seeking actions.

The section for knowledge of danger signs was adopted from a tool by Pembe et al. [5] and is composed of five open-ended questions regarding general knowledge about danger signs during pregnancy, recognition of danger signs, and source of information. Based on the danger signs that a woman can recognize, a list of nine danger signs stated in the WHO guide for essential practice (Childbirth, Postpartum and Newborn Care) [17] was used. These danger signs included the following: (1) severe vaginal bleeding, (2) convulsions, (3) severe headache with blurred vision, (4) severe abdominal pain, (5) too weak to get out of bed, (6) fast or difficulty in breathing, (7) reduced fetal movement, (8) fever, and (9) swelling of the fingers, face, and legs [5]. A woman was considered to *have sufficient knowledge* if she was able to spontaneously mention at least four of the nine danger signs [12]. On the other hand, a woman was considered to *have low knowledge* if she was able to spontaneously mention one to three danger signs, and to *have no knowledge* if she was not able to spontaneously mention any danger sign.

The section for healthcare seeking actions included six questions (forced choice and open-ended) about the recognized danger signs and health actions women had taken for each danger sign. An example of an open-ended question was to explain further why the woman decided to take a particular action after recognizing a danger sign. Visiting a health facility for care was considered as appropriate healthcare seeking action, whereas not doing anything, visiting a traditional healer, self-medication, and going to a traditional birth attendant were considered as inappropriate healthcare seeking actions.

The ANC experience during their last pregnancy involved answering a questionnaire consisting of nine questions (forced choice and open-ended) that queried about the type of care women received during their clinic visits including education and advice.

The tool was pretested in another health center within the municipality to check for clarity. Twenty women of similar status were interviewed and appropriate modifications were made to the questionnaire.

### Data collection

All the women enrolled in this study provided informed consent before participating. Using the questionnaire, the lead researcher and five trained research assistants interviewed women in Swahili at the health facilities. After the interview, the lead researcher collected the questionnaires for data entry and cleaning. There were 384 pregnant women who agreed to participate in the study.

### Data analysis

Descriptive and inferential statistics were used. The participants’ characteristics were evaluated in terms of frequencies. The F-test was used to compare knowledge scores, demographic characteristics, and healthcare seeking actions. The confounding variables that were controlled included educational level, marital status, occupation, parity, gravidity, and ANC visit. A *P*-value <0.05 was considered to indicate a statistically significant difference. Data were analyzed using an SPSS statistical package.

## Results

Of the 392 pregnant women who were eligible, 384 (98%) consented to participate in the study. The participating women responded to all the questions in the questionnaire. Among these 384 women, 67 (17.4%) had experienced danger signs during their pregnancy and reported their healthcare seeking actions after recognizing the danger signs.

### Characteristics of participating women

More than half of the participants (68.8%) were aged 21 to 30 years, and 329 (85.7%) were living with their partners. Among the participants, five (1.3%) had no education, 175 (45.6%) had primary education, 172 (44.8%) had secondary education, and 32 (8.3%) had post-secondary education as their highest educational levels. About half (54.4%; *n* = 209) were either employed or engaged in business which was conducted outside of their home. A total of 374 (97.4%) participants had attended ANC at least once during their last pregnancy, and among these, 271 (70.6%) had visited an antenatal clinic more than four times (Table 1). The gestational age at the first ANC visit was 4 months or more for 204 women (54.4%). Furthermore, 34.9% (*n* = 134) of the respondents were primiparas. About 99% of the participants delivered at a health facility (i.e., hospital, healthcare center, or dispensary). The mean distance to the health facility was 2.4 km (SD = 3.1).

**Table 1:** Characteristics of women and relationship with knowledge about danger signs (N = 384)

Variable	Categories	n (%)	Knowledge Mean score	*F*	*P*
Age	<20	30 (7.8)	1.77	4.05	**0.018**
	21-30	264 (68.8)	2.64		
	>30	90 (23.4)	2.83		
Education level	No education	5 (1.3)	1.40	2.42	0.066
	Primary	175 (45.6)	2.41		
	Secondary	172 (44.8)	2.80		
	Post-secondary	32 (8.3)	2.91		
Marital status	Living with partner	329 (85.7)	2.42	0.759	0.384
	Not living with partner	55 (14.3)	2.65		
Occupation	With occupation	209 (54.4)	2.59	0.419	0.658
	No occupation	163 (42.4)	2.61		
	Student	12 (3.2)	3.08		
Parity	1	160 (41.7)	2.64	0.425	0.654
	2-4	214 (55.7)	2.57		
	≥5	10 (2.6)	3.10		
Gravidity	1	134 (34.9)	2.50	1.032	0.357
	2-4	229 (59.6)	2.64		
	≥5	21 (5.5)	3.10		
ANC visit	<4 visits	113 (29.4)	2.05	1.747	0.187
	≥4 visits	271 (70.6)	2.15		

### Knowledge of danger signs during pregnancy

A total of 335 (87.2%) women reported that they had heard about danger signs during pregnancy. The source of information about the danger signs during pregnancy was from the RCHC for 274 women (81.8%), social gatherings for 58 women (17.4%), and the radio for three women (0.8%).

**Fig. 1:**
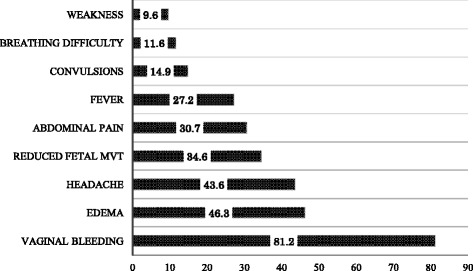
Recall of danger signs during pregnancy (*n* = 335). Vertical axis-Danger signs. Horizontal axis-Percentage

When asked to spontaneously mention the danger signs, more than half of the participants (*n* = 222, 57.8%) were able to mention only one to three danger signs. Only 104 (31%) had correct knowledge of at least four danger signs and nine (2.7%) were not able to mention any item. The mean score for knowledge of danger signs was 3.0 (SD = 1.609). Figure 1 shows the danger signs in ascending frequency. The most commonly known danger signs were vaginal bleeding (81.2%), edema (46.3%), and headache (43.6%).

The rest of the participants (*n* = 49, 12.8%) were not able to spontaneously mention the danger signs. These participants were thus provided with a list of danger signs to help them recall the signs. The most commonly known danger signs in this group were vaginal bleeding (65.3%) and abdominal pain (65.3%).

### Knowledge scores and characteristics of women

There was a significant relationship between age and knowledge of danger signs (*P* = 0.018). The women were classified into three age groups; young age: < 20 years; middle age: 21-30 years; old age: > 31 years. Participants who were older had higher scores than those who were younger (Table 1). The mean score for knowledge of danger signs among women aged 30 years and above was 2.83, whereas that among women aged 20 years and below was 1.77. The other variables, namely, educational level, marital status, occupation, parity, gravidity, and ANC visit were not significantly related to knowledge of danger signs during pregnancy. After performing further analysis and controlling for confounding variables using logistic regression, it was determined that older women were 1.6 times more likely to have knowledge of danger signs than younger women (OR 1.61; 95% CI 1.05-2.46).

### Healthcare seeking actions after recognition of danger signs

The healthcare seeking actions were categorized as either appropriate (visiting a health facility) or inappropriate (taking no action; consulting a friend/relative, or self-medication). A total of 67 (17.4%) participants had recognized danger signs during their last pregnancy. The majority of the women who recognized danger signs (*n* = 61, 91%) went to a health facility for care after experiencing the danger signs. The actions taken for each danger sign experienced are shown in Table 2. All the women who experienced danger signs such as edema and reduced fetal movement went to a health facility for care. The mean score for knowledge of danger signs for those who experienced danger signs was 3.17 (SD = 2.196). For crucial and vivid danger signs such as vaginal bleeding (*n* = 1), convulsions (*n* = 1), and abdominal pain (*n* = 2), some of the women did not visit a health facility for care.

**Table 2:** Healthcare seeking actions for danger signs during pregnancy

Danger signs	Experienced (n = 67)		Total
	Appropriate Action	Inappropriate action	
Vaginal bleeding	34	1	35
Edema	25	0	25
Headache	37	3	40
Reduced fetal movement	24	0	24
Abdominal pain	32	2	34
Fever	26	3	29
Convulsions	23	1	24
Difficulty Breathing	30	1	31
Weakness	34	2	36

Multiple response table. The values are over a total of participants who experienced a particular danger sign

Other danger signs such as fever, headache, and being too weak to get out of bed were dealt with inappropriately because the participants considered these signs as normal events during pregnancy and therefore they decided to either not take any action or buy over-the-counter medicines. Women were asked to further explain why they decided to take the actions they took, and the majority of the participants (*n* = 53, 79.1%) who experienced danger signs explained that they preferred to be treated at the hospital because they believed that their problem would be solved in health facilities. Moreover, seven (10.5%) participants explained that they were educated about the danger signs so they knew that they were supposed to go to the hospital; five women (7.5%) responded that their condition worsened and therefore they had to rush to the hospital to save their lives and their babies. However, two women (3%) explained that the danger signs were normal events during pregnancy and therefore they decided to stay at home.

## Discussion

### Knowledge of danger signs during pregnancy

Our findings indicate that knowledge of danger signs during pregnancy was low among the pregnant women in Tanzania who participated in this study. Slightly less than one-third of the participants were spontaneously able to mention four or more danger signs. Notably, this finding is similar to those of other studies in Africa regarding knowledge of danger signs among pregnant women. In fact, only 26% of women in rural areas in Tanzania were reported to be aware of danger signs during pregnancy [5]. The same trend was also found in a study conducted in Aleta Wondo in Ethiopia, where only 30.4% of the women were aware of at least two danger signs [8].

The most common spontaneously mentioned danger sign of pregnancy was vaginal bleeding (81.2%), possibly because it is the most visible sign compared with other signs such as reduced fetal movement. This finding is similar to that of studies in Ethiopia [8] and Uganda [9] whereby most respondents spontaneously identified vaginal bleeding as a danger sign more than others. However, some previous studies showed a contrasting finding whereby in rural Tanzania only 9.6% and Uganda 49% women were aware of vaginal bleeding as a danger sign during pregnancy [5, 9].

This difference might be due to the study design and location differences as they were all community surveys. Nevertheless, emphasis should also be placed on other danger signs that were not commonly mentioned such as abdominal pain and convulsions. A study by Hailu et al. in Ethiopia revealed that these danger signs were not spontaneously mentioned even though they indicated the presence of (pre-)eclampsia [8]. Only 7% of the participants mentioned abdominal pain and 4.7% convulsion as danger signs.

A significant relationship was found between having knowledge about danger signs and age of the participants. Similar findings were reported from studies in rural Tanzania and South Africa, which found increased awareness among older and multiparous women [5, 10]. Thus, older women have more experience with pregnancy issues. The total fertility rate in Tanzania is 5.4 births per woman. The total fertility rate among rural women on the mainland (6.1 births) is higher than that among urban women (3.7 births) [2]. Therefore, it was likely that they were more aware of danger signs either from their own experience or from events in their society. This implies a need for special consideration among young women, particularly adolescents, when providing health counseling and education at antenatal clinics.

Being young and immature may likely affect the reception of antenatal education and the recognition of signs of obstetric complications. WHO reported that adolescent pregnancy remains a major contributor to maternal mortality and that obstetric complications are the second cause of death among 15 to 19 year olds globally [18]. Most pregnant adolescents lacked social support, experienced community stigmatization, and were treated improperly by health workers [19]. There is therefore a need to introduce and implement special adolescent friendly interventions to empower pregnant adolescents by providing them health information on pregnancy as well as delivery and early childhood care [20]. Pregnant adolescents need to know and be assured that healthcare workers care about them and that they can receive assistance in using the available health facility services. Moreover, techniques such as producing pictorial gifts with health messages that can remind pregnant adolescents of the danger signs during pregnancy and what appropriate actions to take when they recognize a danger sign should be used. Although this study was conducted in an urban district where 97% of women visited an ANC clinic at least once, the present findings suggest that the quality of antenatal health education was poor.

During antenatal health education, a large group of more than 40 women usually gather, with only one or two nurse-midwives handling the hour-long session. The likelihood of some women missing or misinterpreting the educational information provided is higher in a large group than in a small group. A previous study on the quality of antenatal care in rural Tanzania showed that two out of every five women were not counselled on pregnancy danger signs [21]. Regarding the amount of time spent for antenatal care consultation in Tanzania, the mean total duration for the initial ANC consultation was reported to be only about 20 min [22], which is not sufficiently long for proper counselling [11]. These findings imply a poor quality of counselling regarding the danger signs during pregnancy for women who had attended the antenatal clinic. Furthermore, there was an apparent imbalance between demand and supply owing to the overwhelming numbers of women attending the clinics, inadequately skilled staff, and indifferent attitudes of healthcare workers. Nyamtema et al. [11] found substandard ANC in rural Tanzania owing to the lack of staff, equipment, and supplies. The lack of simplicity in the information delivery system for pregnant KwaZulu-Natal women in South Africa [10]. Therefore, considerable and sustained efforts are needed to improve the quality of health education provided at health facilities in Tanzania, aiming at increasing knowledge of danger signs during pregnancy.

### Healthcare seeking action

The present findings revealed that the majority of women who had recognized signs of complications during their pregnancy visited a health facility for care and management. They likely feared for the life of their infant. Also, the majority were living less than five kilometers from the hospital, hence they could easily access the services. They believed that being in a hospital environment could solve most of their health-related concerns.

What was surprising was the decision of some women who experienced danger signs not to take any action. The results showed that those who experienced fever and headache decided to either do nothing or take over-the-counter medicine. These findings in some respects resemble those of a study conducted in Ghana reporting on obstetric danger signs and factors affecting the healthcare seeking behavior which identified a traditional hierarchy of seeking care [23]. For symptoms such as vaginal bleeding, headache, and fever, women usually started with home remedies, progressed to consulting traditional healers, and ended up at a health facility. Furthermore, Kilewo et al. identified perceived delay in healthcare seeking in a study from Bangladesh [24]. They reported that only 33% of the patients sought treatment from a qualified health provider during their pregnancy. More than 75% of the women with time-sensitive complications of convulsions or vaginal bleeding had either failed to seek any treatment or sought treatment from an unqualified provider.

Therefore, factors that affect women’s healthcare seeking should be thoroughly clarified and addressed in the community. Studies have shown that women’s decision to seek care could be greatly influenced by the perceived severity of the condition, distance to the health facility, and financial status to cover hospital bills in case payment was necessary [12, 24]. Furthermore, consideration must be given to the limited decision-making capability of women within marriage and family [4]. Women need to be empowered with knowledge and birth preparedness during the antenatal clinic visit.

### Implications for practice

The present results imply that having knowledge of danger signs is not enough; additional changes in attitude and empowerment to take appropriate action are also required. A lack of educational opportunities and poor understanding of both danger signs and possible complications indicate that many women may not be familiar with the presentation of complications and consider them normal appearances in pregnancy. Delay in seeking appropriate healthcare owing to lack of knowledge of danger signs can be reduced by improving access to health information and education through the development of community outreach projects that specifically provide information on childbearing issues particularly danger signs for obstetric complications. Such information should be given to individual women and their families to facilitate their collaboration when care is needed. The establishment of community-based programs is also of particular importance to assist women with limited ability to visit health facilities. It will also be beneficial if other members of the community receive education and eventually provide a community support group that will offer help when a complication occurs. Importantly, the quality of health education at the health facility should be carefully checked for relevance and usability.

### Limitations and further research

One main limitation of the present study was that the structured interview format limited the ability to explore extensively the reasons for the subsequent actions the women took after recognizing the danger signs and how long it took them to decide to seek care. Future exploratory studies on healthcare seeking behavior will provide insights into the association between having knowledge and subsequent healthcare seeking actions. In addition, the small number of those who recognized danger signs and the use of only two health facilities limit the generalization of our findings. Also, other factors standing in between knowledge and action have not been clearly stated and assessed systematically in the data collection tool. As these other factors were not considered in this study, additional studies are recommended to further assess these factors. Our study was conducted in a clinical setting and in facilities located in an urban district. Therefore, the findings cannot be generalized to women who have failed to attend a clinic soon after delivery and to those living in rural areas. Our sample was biased by the fact that we did not include women who delivered at home as well as women who experienced perinatal deaths, because we sampled after delivery in a clinic where women brought their newborns for immunization.

## Conclusion

This is apparently the first study that assessed the knowledge of danger signs during pregnancy and subsequent healthcare seeking actions of women in urban Tanzania. The findings indicate their low knowledge of danger signs during pregnancy and provide important insights into the possible underlying factors. Older participants had higher scores for knowledge of danger signs than younger participants. Women’s knowledge of danger signs during pregnancy positively influenced their decisions regarding when to seek medical care and when to take appropriate action. Further studies are recommended to address the knowledge gap and to plan more effective interventions for improving antenatal care in limited resource settings.

## Abbreviations

ANC: Antenatal Care
FANC: Focused Antenatal Care
IRB: Institutional Review Board
MUHAS: Muhimbili University of Health and Allied Sciences
RCHC: Reproductive and Child Health Clinic
WHO: World Health Organization
